# Quantitative proteomic, physiological and biochemical analysis of cotyledon, embryo, leaf and pod reveals the effects of high temperature and humidity stress on seed vigor formation in soybean

**DOI:** 10.1186/s12870-020-02335-1

**Published:** 2020-03-26

**Authors:** Jiaping Wei, Xiaolin Liu, Linzhi Li, Haihong Zhao, Sushuang Liu, Xingwang Yu, Yingzi Shen, Yali Zhou, Yajing Zhu, Yingjie Shu, Hao Ma

**Affiliations:** 1 State Key Laboratory of Crop Genetics and Germplasm Enhancement, Nanjing Agricultural University, Nanjing, 210095 China; 2 Crop and Soil Sciences Department, North Carolina State University, Raleigh, NC 27695 USA; 3 College of Agriculture, Anhui Science and Technology University, Fengyang, 233100 China

**Keywords:** Soybean, High temperature and humidity stress, Seed vigor, Proteomic, Ultrastructure, Physiology and biochemistry

## Abstract

**Background:**

Soybean developing seed is susceptible to high temperature and humidity (HTH) stress in the field, resulting in vigor reduction. Actually, the HTH in the field during soybean seed growth and development would also stress the whole plant, especially on leaf and pod, which in turn affect seed growth and development as well as vigor formation through nutrient supply and protection.

**Results:**

In the present study, using a pair of pre-harvest seed deterioration-sensitive and -resistant cultivars Ningzhen No. 1 and Xiangdou No. 3, the comprehensive effects of HTH stress on seed vigor formation during physiological maturity were investigated by analyzing cotyledon, embryo, leaf, and pod at the levels of protein, ultrastructure, and physiology and biochemistry. There were 247, 179, and 517 differentially abundant proteins (DAPs) identified in cotyledon, embryo, and leaf of cv. Xiangdou No. 3 under HTH stress, while 235, 366, and 479 DAPs were identified in cotyledon, embryo, and leaf of cv. Ningzhen No. 1. Moreover, 120, 144, and 438 DAPs between the two cultivars were identified in cotyledon, embryo, and leaf under HTH stress, respectively. Moreover, 120, 144, and 438 DAPs between the two cultivars were identified in cotyledon, embryo, and leaf under HTH stress, respectively. Most of the DAPs identified were found to be involved in major metabolic pathways and cellular processes, including signal transduction, tricarboxylic acid cycle, fatty acid metabolism, photosynthesis, protein processing, folding and assembly, protein biosynthesis or degradation, plant-pathogen interaction, starch and sucrose metabolism, and oxidative stress response. The HTH stress had less negative effects on metabolic pathways, cell ultrastructure, and physiology and biochemistry in the four organs of Xiangdou No. 3 than in those of Ningzhen No. 1, leading to produce higher vigor seeds in the former.

**Conclusion:**

High seed vigor formation is enhanced by increasing protein biosynthesis and nutrient storage in cotyledon, stronger stability and viability in embryo, more powerful photosynthetic capacity and nutrient supply in leaf, and stronger protection in pod under HTH stress. These results provide comprehensive characteristics of leaf, pod and seed (cotyledon and embryo) under HTH stress, and some of them can be used as selection index in high seed vigor breeding program in soybean.

## Background

Temperature and humidity are two of the pivotal environmental factors associated with seed growth and development in field. High temperature and humidity condition can not only affect seed yield and quality, but also reduce seed vigor and storage capacity [1, 2], resulting in the pre-harvest seed deterioration. Seed vigor is a complex property that determines the seed’s potential for rapid uniform germination and subsequent growth [3]. Moreover, HTH stress can also seriously interfere with the membrane compositions during seed development [4].

Soybean (*Glycine max* (L.) Merrill) is one of the most important legume crops and has a major impact on the global economies [5]. The vigor formation in developing soybean seeds generally starts from physiological maturity (R7) period. During this period, the developing seeds are susceptible to HTH stress, leading to the reduction of vigor. This situation occurs in many soybean production areas around the world [6–8]. Proteins are important structural components of cytoskeleton, membranes and cell wall in plants, which are responsible for most metabolic pathways and cellular processes in the seed. Hence, it would make senses to understand plant physiological processes by describing the proteome of a seed, a seed tissue, a specific cell type or a subcellular compartment [9].

So far, many researchers have investigated seed vigor at protein level [10–20]. Recently, several proteomic studies have focused on the effects of HTH stress on the vigor formation of developing soybean seed. For example, Wang et al. [21] analyzed the effects of HTH stress on the vigor formation of a pre-harvest seed deterioration-sensitive soybean cultivar by two-dimensional electrophoresis (2-DE). Ma et al. [22] revealed the impacts of HTH stress on the vigor formation of a pre-harvest seed deterioration-resistant soybean cultivar by 2-DE. Song et al. [23] reported a differentially proteomic analysis of developing seeds using a pair of pre-harvest seed deterioration-sensitive and -resistant soybean cultivars under HTH stress. In these studies, many proteins were found to be involved in the metabolic pathways and cellular processes that are potentially related to soybean seed vigor. Moreover, the pre-harvest deterioration-resistant cultivar possessed greater ability of ROS scavenging and cell defensing compared to the pre-harvest deterioration-sensitive cultivar under HTH stress. However, all these studies used the whole developing seeds as experimental materials to investigate the effects of HTH stress on soybean seed vigor formation. Actually, besides the developing seed, HTH condition would affect the whole plant, especially on leaf and pod. Soybean leaf is the primary site of photosynthesis, which contributes to the biosynthesis of plant biomass and energy [24, 25]. Soybean pod skin not only protects seeds, but also provides nutrients for seed growth and development through photosynthesis. Thus, it is imperative to consider the responses of leaf and pod when investigating seed vigor formation under HTH stress. In the present study, the comprehensive effects of HTH stress on seed vigor formation were evaluated using a pair of pre-harvest seed deterioration-sensitive and -resistant soybean cultivars at the levels of protein, ultrastructure, and physiology and biochemistry. The technique of isobaric tags for relative and absolute quantification (iTRAQ) was employed to detect the changes of proteins during stress. The aims are to find the major metabolic pathways and cellular processes involved in seed vigor formation in cotyledon, embryo, leaf and pod, and to lay a foundation for further revealing the mechanism of seed vigor formation under HTH stress.

## Results

### Physiological responses to HTH stress

**Fig. 1:**
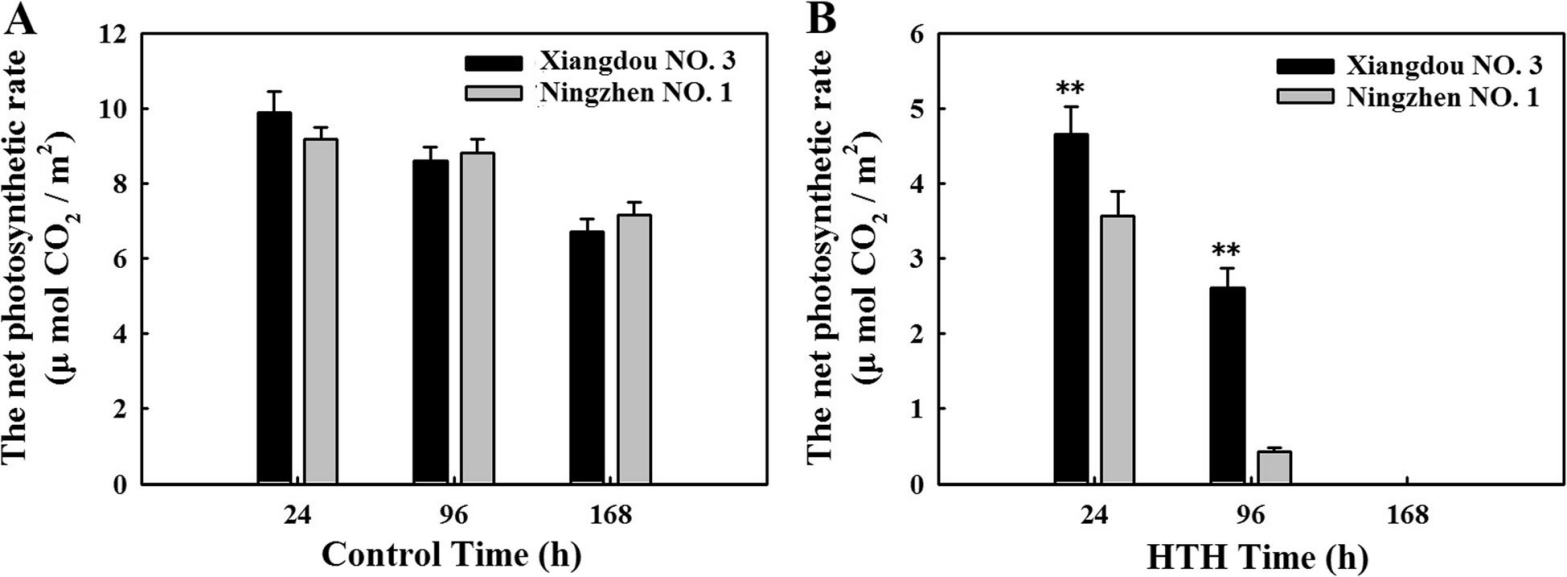
The net photosynthetic rates of soybean cvs. Xiangdou No. 3 and Ningzhen No. 1. **a**, under the control condition; **b**, under the HTH stress. Values shown are means ±SD from three biological replicates (**, *p* < 0.01)

No differences in net photosynthetic rates were found between the pre-harvest seed deterioration-resistant soybean cv. Xiangdou No. 3 and -sensitive cv. Ningzhen No. 1 under the normal condition (Fig. 1a). However, notably, the net photosynthetic rates of soybean cv. Xiangdou No. 3 were significantly (*p* < 0.01) higher than those of cv. Ningzhen No. 1 at the stress time points of 24 and 96 h (Fig. 1b).

### Microstructure comparison under HTH stress

**Fig. 2:**
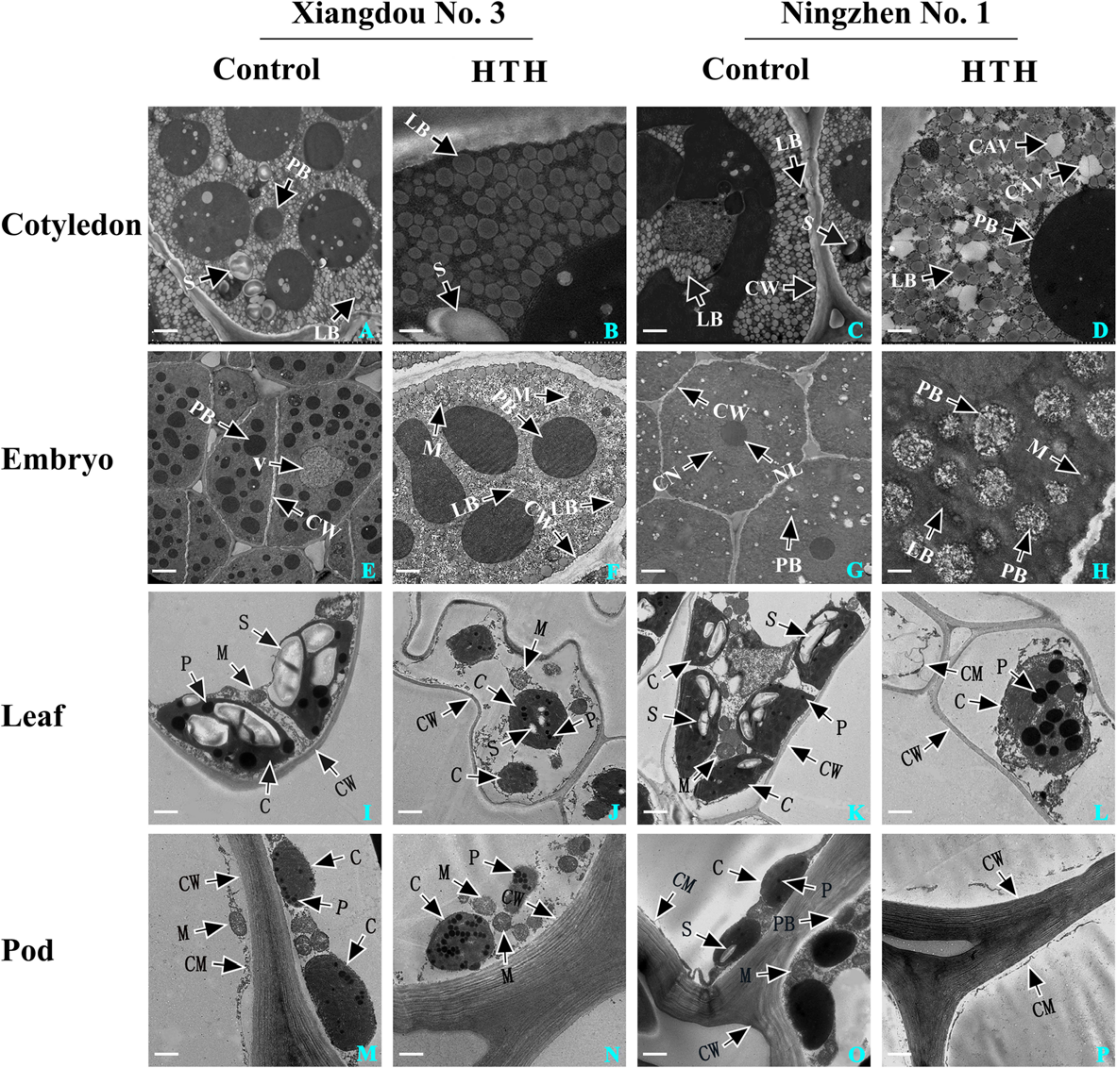
Transmission electron micrographs of soybean cvs. Xiangdou No. 3 and Ningzhen No. 1. Microstructure changes in cotyledon (A, B, C, D), embryo (E, F, G, H), leaf (I, J, K, L) and pod (M, N, O, P) of both the cultivars under the HTH stress (96 h) and control (96 h). A, C, I, K, 5.0 μm; E, G, J, L, 10.0 μm; B, D, 1.0 μm; F, H, M-P, 2.0 μm; C, chloroplast; CAV, cavitation; CW, cell wall; CM, cell membrane; CN, cell nucleus; LB, lipid body; M, mitochondrion; P, phagosome; PB, protein body; S, starch grain

Transmission electron microscope (TEM) analysis was conducted using the cotyledons, embryos, leaves, and pods of two cultivars under the HTH stress (at 96 h) and control (at 96 h), respectively. Compared to the control (Fig. 2A), no obvious cyto-architecture changes were found in the cotyledon cell of cv. Xiangdou No. 3 (Fig. 2B). However, in the cotyledon cell of cv. Ningzhen No. 1, lipid bodies were found to be loosely arranged and a lot of cavities appeared under the HTH stress (Fig. 2C and D). Compared to the control (Fig. 2E), the number of protein bodies in the embryo cell of cv. Xiangdou No. 3 were accordingly reduced (Fig. 2F), while a larger number of protein bodies were disintegrated in the embryo cell of cv. Ningzhen No. 1 under the HTH stress (Fig. 2G and H). The number of chloroplasts and starch grains in plant mesophyll cell were found to be decreased severely in both the soybean cultivars under the HTH stress, and furthermore, more severe decrease was found in soybean cv. Ningzhen No. 1 than in cv. Xiangdou No. 3 (Fig. 2I and J, Fig. 2K and L). In addition, a large number of phagosomes were observed in cv. Ningzhen No. 1 under the HTH stress, indicating that the senescence of its leaf cell was accelerated by stress (Fig. 2 K and L). In the pod (skin) cells, compared to the control (Fig. 2M), the number of mitochondrion in cv. Xiangdou No. 3 was increased after HTH stress (Fig. 2N). Interestingly, compared to the control (Fig. 2O), all the organelles in the pod (skin) cells of cv. Ningzhen No. 1 were disappeared under the HTH stress (Fig. 2P). All these results indicated that the HTH stress exerts greater negative effects on the leaf, pod, cotyledon and embryo of soybean cv. Ningzhen No. 1 than on those of cv. Xiangdou No. 3.

### Change of soluble protein, soluble sugar, malondialdehyde (MDA), starch contents under HTH stress

**Fig. 3:**
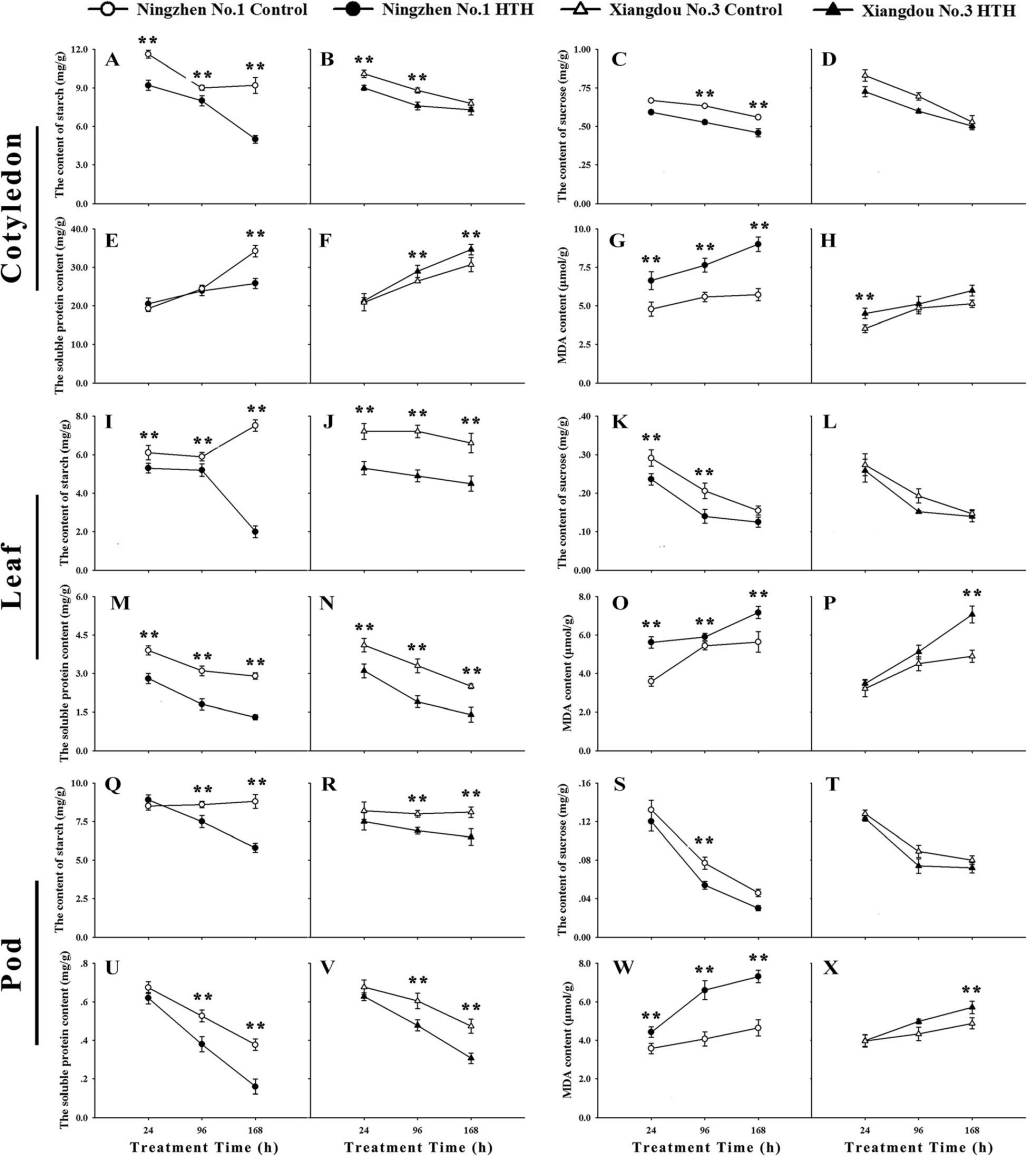
Physiological measurement in soybean cvs. Xiangdou No. 3 and Ningzhen No. 1 under HTH stress. Starch content in cotyledon (A, B), leaf (I, J) and pod (Q, R); Sucrose content in cotyledon (C, D), leaf (K, L) and pod (S, T); Soluble protein content in cotyledon (E, F), leaf (M, N) and pod (U, V); MDA content in cotyledon (G, H), leaf (O, P) and pod (W, X); Values shown are means ± SD from three biological replicates. (**, *p* < 0.01)

The contents of soluble protein, soluble sugar, MDA and starch in the cotyledon, leaf, and pod (skin) of both the soybean cultivars were investigated, respectively (Fig. 3). The contents of starch (Fig. 3A and B) and sucrose (Fig. 3C and D) were decreased in the cotyledon of both the cultivars under HTH stress, but the starch content of cv. Xiangdou No. 3 maintained higher level than that of cv. Ningzhen No. 1 at the stress time point of 168 h. The soluble protein content in the cotyledon was decreased in cv. Ningzhen No. 1 but increased in cv. Xiangdou No. 3 under the HTH stress (Fig. 3E and F). In addition, the MDA content was increased in the cotyledon of both the cultivars under the HTH stress, but the MDA content in the cotyledon of cv. Ningzhen No. 1 maintained much higher level than in that of cv. Xiangdou No. 3 (Fig. 3G and H). The HTH stress caused significant decrease in the starch (Fig. 3I and J, Fig. 3Q and R), sucrose (Fig. 3K and L, Fig. 3S and T) and soluble protein (Fig. 3M and N, Fig. 3U and V) contents in the leaf and pod of both the soybean cultivars. However, the starch (Fig. 3J and R) and soluble protein (Fig. 3N and V) contents in the leaf and pod, and the sucrose (Fig. 3L and T) contents in the pod of cv. Xiangdou No. 3 maintained higher level than those of cv. Ningzhen No. 1 at the stress time point of 168 h. The contents of MDA were found to be increased in the leaf and pod of both the soybean cultivars under the HTH stress, but the MDA contents of cv. Ningzhen No. 1 were higher than those of cv. Xiangdou No. 3 (Fig. 3O and P, Fig. 3W and X).

The enzyme activities of almost all peroxidase (POD), superoxide dismutase (SOD) and catalase (CAT) in the cotyledon, embryo, leaf, and pod of cv. Xiangdou No. 3 were found to be significantly (*p* < 0.05 or *p* < 0.01) increased under the HTH stress. However, the activities of most the enzymes were significantly (*p* < 0.05 or *p* < 0.01) reduced in cv. Ningzhen No. 1 under the HTH stress (Additional file 1: Fig. S1; Additional file 2: Fig. S2; Additional file 3: Fig. S3; Additional file 4: Fig. S4). These results indicated that the reactive oxygen species (ROS) scavenging capacity of soybean cv. Xiangdou No. 3 was stronger than that of cv. Ningzhen No. 1 under the HTH stress.

### Effect of HTH stress on germination of soybean seeds

**Fig. 4:**
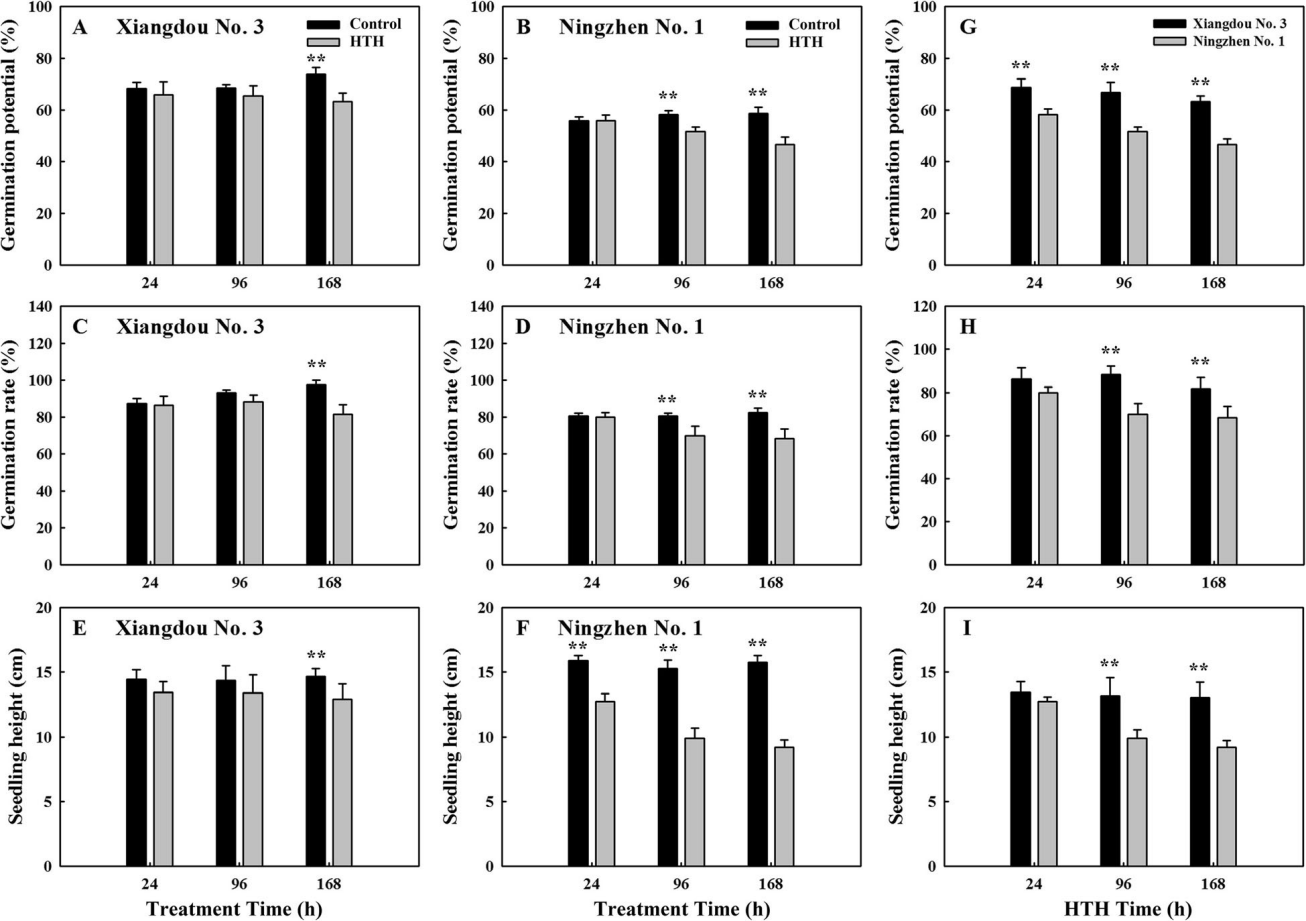
Analysis of germination potential, germination rate and seedling height. The germination potential of pre-harvest seed deterioration-resistant soybean cv. Xiangdou No. 3 (**a**) and -sensitive cv. Ningzhen No. 1 (**b**) under the HTH stress and control. The germination rate of cv. Xiangdou No. 3 (**c**) and cv. Ningzhen No. 1 (**d**) under the HTH stress and control. The seedling height of cv. Xiangdou No. 3 (**e**) and cv. Ningzhen No. 1 (**f**) under the HTH stress and control. The germination potential (**g**), germination rate (**h**) and seedling height (**i**) of cv. Xiangdou No. 3 and cv. Ningzhen No. 1 under the HTH stress. Values are means ± SD from three biological replicates (**, *p* < 0.01)

Compared to the controls, the germination potential, germination rate and seedling height of soybean cv. Xiangdou No. 3 were not significantly (*p* > 0.05) changed at the HTH stress time points of 24 and 96 h, but markedly (*p* < 0.01) reduced at 168 h (Fig. 4a, c and e). The germination potential and germination rate of soybean cv. Ningzhen No. 1 were significantly (*p* < 0.01) decreased at the stress time points of 96 and 168 h (Fig. 4b and d). The seedling height of soybean cv. Ningzhen No. 1 was significantly (*p* < 0.01) decreased at the stress time points of 24, 96 and 168 h (Fig. 4f). Moreover, the germination potential, germination rate, and seedling height of soybean cv. Ningzhen No. 1 were more significantly (*p* < 0.01) decreased than those of cv. Xiangdou No. 3 under the HTH stress (Fig. 4g, h and i). These results showed that soybean cv. Xiangdou No. 3 possessed higher seed germination vigor than cv. Ningzhen No. 1 under the HTH stress.

### Quantitative proteomic analysis in cotyledon, embryo, and leaf under HTH stress

**Fig. 5:**
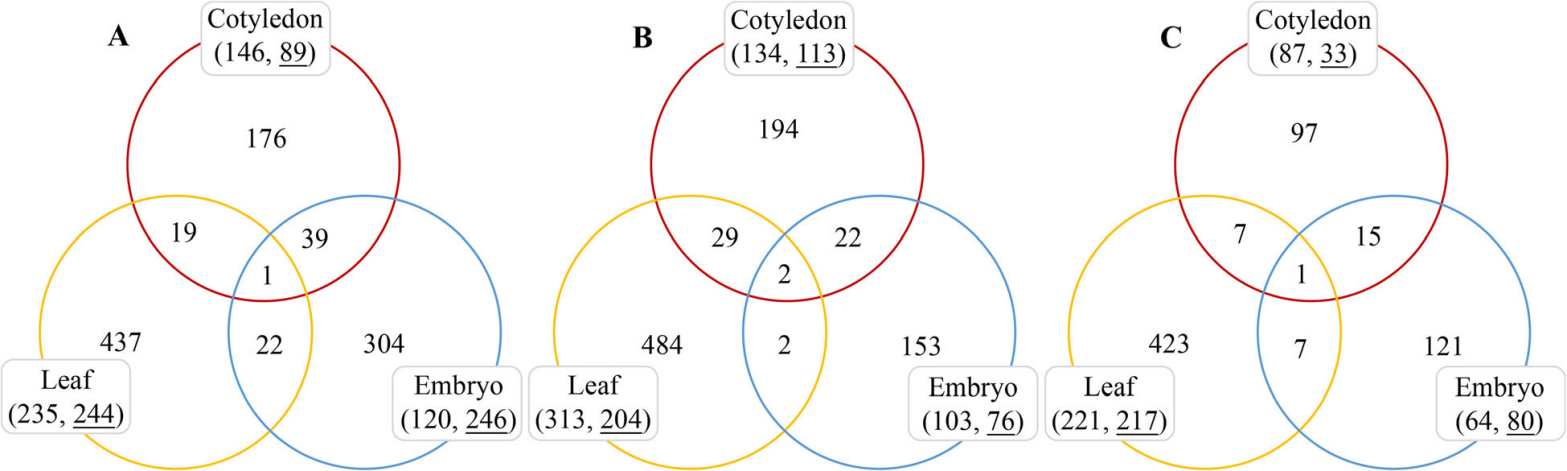
Venn diagram analysis of the number of DAPs. DAPs in leaf, cotyledon, and embryo between the control and HTH stress in soybean cv. Ningzhen No. 1 (**a**) and cv. Xiangdou No. 3 (**b**), respectively. DAPs in leaf, cotyledon, and embryo between both the cultivars under HTH stress (**0063**). The numbers without underline in brackets represent the DAPs accumulated in abundance, while the numbers with underline in brackets represents the DAPs reduced in abundance

All the above results indicated that soybean cv. Xiangdou No. 3 was more tolerant to HTH stress than cv. Ningzhen No. 1 during seed growth and development. Therefore, the cotyledons, embryos and leaves from the stressed and control plants (R7 period) were sampled at 24, 96, and 168 h during the treatment, respectively, for iTRAQ analysis. In soybean cv. Ningzhen No. 1, a total of 235, 366 and 479 DAPs were identified in cotyledon, embryo and leaf under the HTH stress, respectively. Among them, 146 proteins in cotyledon, 120 proteins in embryo, and 235 proteins in leaf were found to be accumulated in abundance, whereas 89 proteins in cotyledon, 246 proteins in embryo, and 244 proteins in leaf were reduced in abundance (Fig. 5a; Additional file 5: S1-S3).

In soybean cv. Xiangdou No. 3, a total of 247, 179, and 517 DAPs were identified in cotyledon, embryo and leaf under the HTH stress, respectively. Among them, 134 proteins in cotyledon, 103 proteins in embryo, and 313 proteins in leaf were found to be accumulated in abundance, while 113 proteins in cotyledon, 76 proteins in embryo, and 204 proteins in leaf were reduced in abundance (Fig. 5b; Additional file 5: S4-S6).

The proteome profiles of corresponding organs and HTH stress points were compared between the soybean cv. Xiangdou No. 3 and cv. Ningzhen No. 1. A total of 120, 144, and 438 DAPs were identified in the cotyledon, embryo and leaf, respectively. Among them, the abundances of 87 proteins in cotyledon, 64 proteins in embryo, and 221 proteins in leaf were found to be accumulated. In addition, the abundances of 33 proteins in cotyledon, 80 proteins in embryo, and 217 proteins in leaf were reduced (Fig. 5c; Additional file 5: S7- S9).

### GO and KEGG analysis of the DAPs under HTH stress

The identified DAPs were further subjected to GO classification and KEGG pathway analysis. There were 42 metabolic pathways in the cotyledon and 50 in embryo altered in the pre-harvest seed deterioration-sensitive soybean cv. Ningzhen No. 1, while 37 in the cotyledon and 41 in the embryo were changed in the pre-harvest seed deterioration-resistant cv. Xiangdou No. 3 under HTH stress. Among them, the metabolic process, cytoplasm and enzyme regulator activity were the largest groups in the biological process, cellular component and molecular function categories, respectively, in the cotyledon and embryo of cv. Ningzhen No. 1 and in the embryo of cv. Xiangdou No. 3 (Additional file 7: Fig. S7 A; Additional file 8: Fig. S8 AB; Additional file 6: S1, S2, S5). The biosynthesis of terpenoids was enhanced, whereas the sucrose and starch metabolism was reduced in the cotyledon and embryo of cv. Ningzhen No. 1 (Additional file 6: S1, S2). In the cotyledon of cv. Xiangdou No. 3, the response to stimulus, cytoplasm and binding were the largest group in the biological process, cellular component and molecular function categories, respectively (Additional file 7: Fig. S7 B; Additional file 6: S4). The arginine biosynthesis was enhanced in the cotyledon and embryo of cv. Xiangdou No. 3 (Additional file 6: S4, S5). There were 53 and 57 metabolic pathways altered in the leaf of cvs Ningzhen No. 1 and Xiangdou No. 3, respectively. Among them, the metabolic process, chloroplast and binding were ranked the first in the biological process, cellular component and molecular function categories, respectively, in cv. Ningzhen No. 1 (Additional file 9: Fig. S9 A; Additional file 6: S3), whereas the metabolic process, chloroplast and enzyme regulator activity were ranked the first in the biological process, cellular component and molecular function categories, respectively, in cv. Xiangdou No. 3 (Additional file 9: Fig. S9 B; Additional file 6: S6). The protein processing in endoplasmic reticulum, tricarboxylic acid (TCA) cycle and fatty acid degradation were enhanced, while the photosynthesis, signal transduction and plant pathogen interaction were reduced in the leaf of cv. Ningzhen No. 1 (Additional file 6: S3). The TCA cycle, pyruvate metabolism and sulfur metabolism were enhanced, whereas the photosynthesis and plant pathogen interaction were reduced in the leaf of cv. Xiangdou No. 3 (Additional file 6: S6).

For between soybean cv. Xiangdou No. 3 and cv. Ningzhen No. 1 under the HTH stress, there were 26 and 29 metabolic pathways altered in the cotyledon and embryo, respectively. Among them, in cotyledon, the metabolic process, chloroplast and enzyme regulator activity were the largest groups in the biological process, cellular component and molecular function categories, respectively (Additional file 7: Fig. S7 C; Additional file 6: S7); in the embryo, the translation, cytoplasm and nutrient reservoir activity were the largest groups in the biological process, cellular component and molecular function categories, respectively (Additional file 8: Fig. S8 C; Additional file 6: S8). Moreover, in the cotyledon, the abscisic acid signaling, cytoskeleton, protein biosynthesis and oxidative stress response were enhanced under the HTH stress (Additional file 6: S7), while in the embryo, the abscisic acid signaling and oxidative stress response were enhanced under the HTH stress (Additional file 6: S8). There were 62 metabolic pathways found to be altered in the leaf. Among them, the metabolic process, chloroplast and enzyme regulator activity were ranked the first in the biological process, cellular component and molecular function categories, respectively (Additional file 9: Fig. S9 C; Additional file 6: S9). In addition, the arginine biosynthesis, carbon fixation, photosynthesis, TCA cycle, glutathione metabolism, and sulfur metabolism were enhanced, while the fatty acid degradation and phosphatidylinositol signaling were reduced (Additional file 6: S9).

### Transcriptional analysis of DAPs

**Fig. 6:**
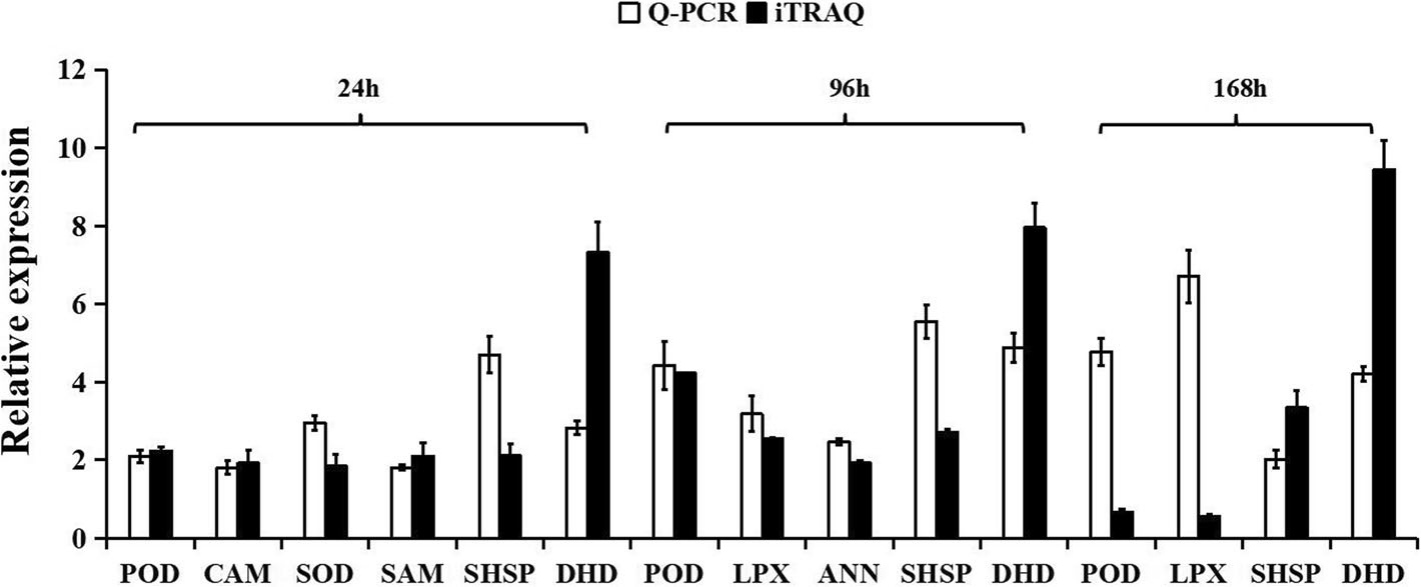
Comparative analysis of mRNA and protein levels of selected genes encoding the differentially abundant proteins identified in seeds between soybean cv. Xiangdou No. 3 and cv. Ningzhen No. 1 under HTH stress. Values are means ± SD (*n* = 3) through comparison of cv. Xiangdou No. 3 to cv. Ningzhen No. 1. Annexin, ANN; Calmodulin, CMD; Dehydrin, DHD; Lipoxygenase, LPX; Peroxidase, POD; Stress-induced protein SAM22, SAM; Superoxide dismutase, SOD; Small heat shock protein, chloroplastic, sHSP

The quantitative RT-PCR (qRT-PCR) analysis was used to validate the expressions of eight genes encoding candidate HTH-responsive proteins identified in the developing seeds between soybean cv. Xiangdou No. 3 and cv. Ningzhen No. 1 under HTH stress by iTRAQ analysis. Six genes were found to be increased consistently at both the mRNA and protein levels under HTH stress in soybean cv. Xiangdou No. 3 compared to cv. Ningzhen No. 1. These six genes encode annexin (ANN), small heat shock protein (SHSP), SOD, dehydrin (DHD), stress-induced protein SAM22 (SAM), and camodulin (CAM), respectively. Besides, two genes encoding POD and lipoxygenase (LPX) showed inconsistency between the mRNA and protein levels (Fig. 6). This discrepancy might be attributed to posttranscriptional and posttranslational regulatory processes [26].

## Discussion

High temperature and humidity are two pivotal factors that result in the decrease of soybean seed vigor during seed growth and development in the field, which is common in many soybean production areas around the world [6–8]. Recently, some studies have focused on the effects of HTH stress on the vigor formation of soybean developing seed using proteomic technologies [21–23], however, all these studies used the whole developing seed as experimental materials. In the present study, a comprehensive investigation was performed to reveal the effects of HTH stress on soybean seed vigor formation at levels of proteins, ultrastructure, and physiology and biochemistry using cotyledon, embryo, leaf, and pod.

### Comparison of effects of HTH stress on seed vigor formation between the two soybean cultivars

**Fig. 7:**
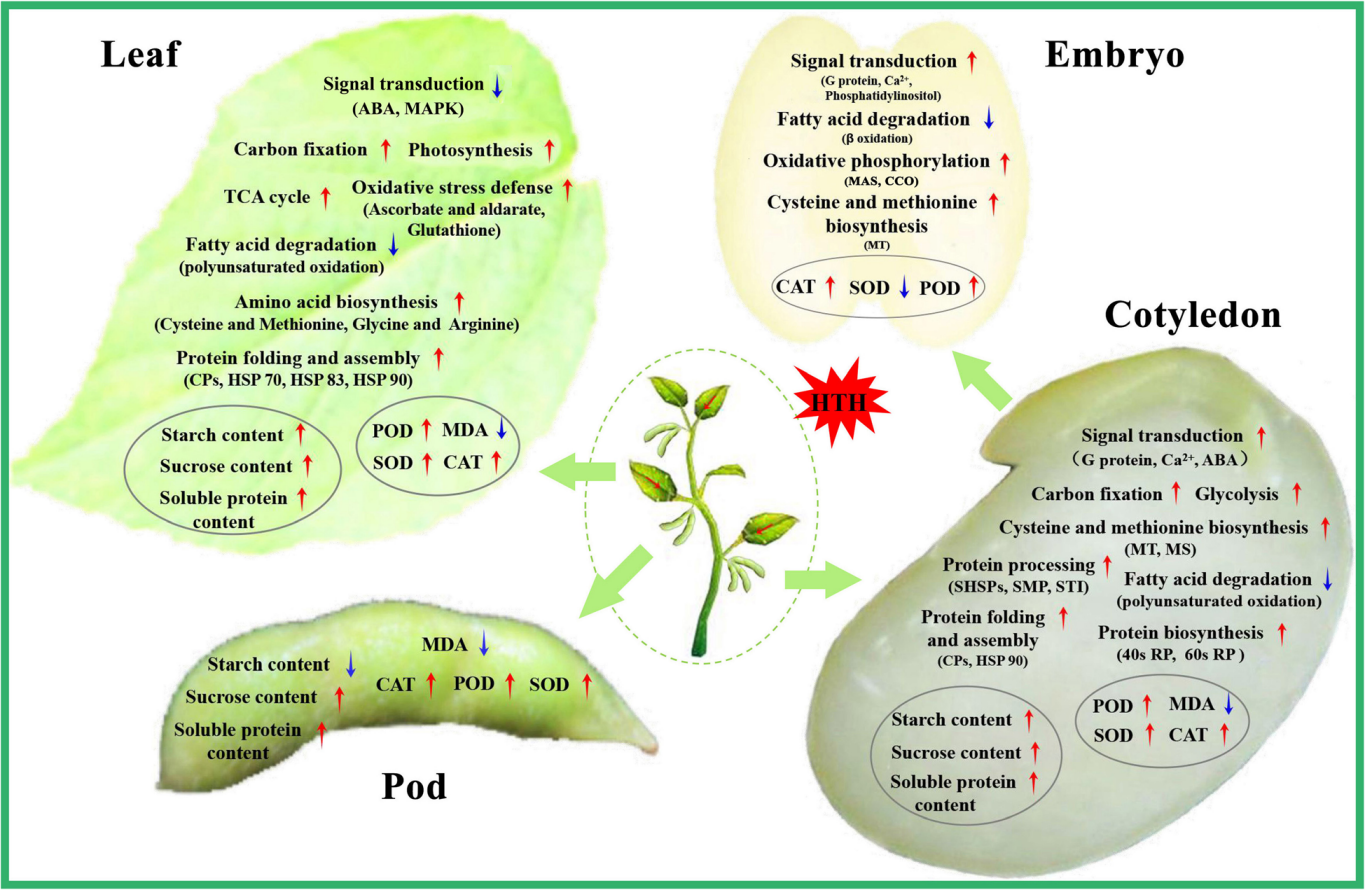
Effects of HTH stress on physiological and biochemical processes in soybean. The major changed metabolic pathways, cellular processes and metabolites contents in leaf, pod skin, cotyledon, and embryo between the two cultivars under HTH stress. The positive metabolic pathways, cellular processes and metabolites are marked with “↑”, while the negativeis marked with “↓”. CAT, catalase; CP, chaperone protein; HTH, high temperature and humidity; MDA, malondialdehyde; MT, 5-methyltetrahydropteroyltriglutamate-homocysteine methyltransferase; MS, methionine synthase; POD, peroxidase; SHSP, small heat shock protein; SMP, seed maturation protein PM22; SOD, superoxide dismutase; STI, heat shock protein STI; RP, ribosomal protein

In the cotyledon, the signal pathways [abscisic acid (ABA)-mediated, Ca^2+^-mediated, and G protein-mediated], carbon fixation [ribulose bisphosphate carboxylase small chain (RuBisCO)], glycolysis [glyceraldehyde-3-phosphate dehydrogenase (GAPDH), alcohol dehydrogenase (ADH)], cysteine and methionine biosynthesis [5-methyltetrahydropteroyltriglutamate- homocysteine methyltransferase (MT), methionine synthase (MS)], protein biosynthesis [40S ribosomal protein (RP), 60S RP], protein processing [heat shock protein STI (STI), SHSPs, SMP], and protein folding and assembly [chaperone protein (CPs), HSP 90] were enhanced, while the fatty acid degradation (polyunsaturated oxidation) was decreased only in soybean cv. Xiangdou No. 3 compared to cv. Ningzhen No. 1 under the HTH stress (Fig. 7; Additional file 10: Table S10). The increased protein biosynthesis in the cotyledon of soybean cv. Xiangdou No. 3 under the HTH stress was consistent with the changes of soluble protein contents (Fig. 7). Yin et al. [18] reported that ABA is involved in seed aging. Similar results in this study indicated that soybean cv. Xiangdou No. 3 may resist HTH stress by enhancing ABA signaling that may be caused by elevating endogenous ABA levels. RuBisCO is a key enzyme in Calvin cycle and plays a central role in the carbon fixation of photosynthesis. It catalyzes the carboxylation of ribulose-1,5-bisphosphate to yield two molecules of 3-phosphoglycerate, and simultaneously oxidizes the pentose substrate in the photorespiration process [27]. It has been reported that soybean seed at physiological maturity stage contains some mature chloroplasts without any photosynthesis activity [21]. Therefore, the accumulated RuBisCO implies that the photorespiration rate might be raised in the cotyledon of soybean cv. Xiangdou No. 3 in response to the HTH stress. Hirokazu et al. [28] showed that a reduction in ADH reduced seedling viability and decreased sugar concentrations in the rice seed. Additionally, *AtADH1* overexpressing plants accumulated higher levels of total soluble sugars and sucrose than WT plants under control and stress conditions [29]. In this study, the accumulated ADH was accompanied with high levels of soluble proteins, sucrose and starch (Fig. 3), implying that the effects of ADH on seed vigor might be directly related to the enhanced nutrient storage. MT and MS catalyze the terminal step of de novo biosynthesis of methionine, which is an intermediate in the biosynthesis of polyamines, ethylene and biotin [30]. Increased biotin synthesis proteins are crucial for cell membrane stability under HTH stress [21]. In addition, Yacoubi et al. [12] reported that accumulated MT increased in parallel seed vigor, which is consistent with our TEM analysis and proteomic results (Fig. 2; Fig. 7). It has been reported that RPs perform independent functions of protein biosynthesis and the amounts of HSPs and CPs are all correlated closely with seed vigor. Catusse et al. [11] reported that the GAPDH and RPs were correlated positively with seed vigor in sugarbeet. Wu et al. [31] suggested that the HSPs were more abundant in high vigor maize seeds. Rajjou et al. [10] showed that GAPDH was increased, while CPs were inhibited in the artificially aged *Arabidopsis* seeds. In this study, our results showed that high levels of CAT, POD, SOD activities and low level of MDA content in the cotyledon of soybean cv. Xiangdou No. 3 under the HTH stress (Additional file 1: Fig. S1; Fig. 3), indicating that the function of cotyledon especially in cell ultrastructure stability, nutrient storage, and protein biosynthesis in pre-harvest seed deterioration-resistant soybean cv. Xiangdou No. 3 was stronger than in pre-harvest seed deterioration-sensitive soybean cv. Ningzhen No. 1 under HTH stress.

In the embryo, the signal pathways (G protein-mediated, and Ca^2+^-mediated, phosphatidylinositol), oxidative phosphorylation [Mitochondrial ATP synthase subunit O (MAS), cytochrome c oxidase (CCO)], and cysteine and methionine biosynthesis (MT) were enhanced and the ABA signaling and fatty acid degradation (β oxidation) were reduced only in soybean cv. Xiagndou No. 3 compared to cv. Ningzhen No. 1 under the HTH stress (Fig. 7; Additional file 10: Table S10). ATP synthase subunit is a membrane-bound enzyme complexes transporter that combines ATP synthesis or hydrolysis with the transport of protons across the inner mitochondrial membrane [32]. CCO is the last complex IV of electron transfer chain in mitochondria and responsible for transferring electrons from oxidized cytochrome c to the final acceptor oxygen [33]. The accumulated MAS and CCO imply the increase of energy requirement during seed developing under HTH stress. Our previous seed viability test had proved that the soybean cv. Xiangdou No. 3 had stronger viability than cv. Ningzhen No. 1 under HTH stress [34]. Compared to a previous research [23], the phosphatidylinositol signaling was a novel finding, and it was increased in the embryo of soybean cv. Xiagndou No. 3 under the HTH stress in contrast to cv. Ningzhen No. 1. Phosphatidylinositol is a precursor structural phospholipid composed primarily of phosphorus, which can produce compounds involved in membrane integrity [35, 36]. We speculated that the enhanced phosphatidylinositol signaling might maintain the stability of embryo cells in soybean cv. Xiagndou No. 3 under the HTH stress. Combined with the results of TEM, physiology and biochemistry (Fig. 2; Additional file 2: Fig. S2), our results indicated that the embryo of pre-harvest seed deterioration-resistant soybean cv. Xiangdou No. 3 had stronger viability than that of pre-harvest seed deterioration-sensitive soybean cv. Ningzhen No. 1 under HTH stress.

The photosynthesis, carbon fixation, TCA cycle, oxidative stress defense, amino acid biosynthesis (cysteine, methionine, glycine, arginine), and protein folding and assembly (CPs, HSP 70, HSP 83, HSP 90) were enhanced, while the signaling [ABA, mitogen-activated protein kinase (MAPK)] and fatty acid degradation (polyunsaturated oxidation) were reduced in the leaf of cv. Xiangdou No. 3, compared to that of cv. Ningzhen No. 1 under the HTH stress (Fig. 7; Additional file 10: Table S10). Here, the increased oxidative stress defense (ascorbate and aldarate, glutathione metabolism) in the leaf of soybean cv. Xiangdou No. 3 under the HTH stress compared to cv. Ningzhen No. 1 was consistent with the results of antioxidant enzymes activities (Fig. 7). ABA and MAPK play a pivotal role in transduction of diverse extracellular stimuli such as biotic and abiotic stresses as well as a range of developmental responses including differentiation, proliferation and death [19, 37]. In this study, MAPK signaling was decreased in the leaf of soybean cv. Xiangdou No. 3 under the HTH stress, compared to soybean cv. Ningzhen No. 1, implying that cell death might be enhanced in the leaf of soybean cv. Ningzhen No. 1 under HTH the stress. Interestingly, the reduction of zeaxanthin epoxidase for ABA synthesis might suggest the existence of feedback regulation (Fig. 7; Additional file 6: Table S9). Consistent with proteomic results, the measured net photosynthetic rates were significantly (*p* < 0.01) higher in soybean cv. Xiangdou No. 3 than in cv. Ningzhen No. 1 under the HTH stress, which might attribute to more chloroplasts in the leaves of soybean cv. Xiangdou No. 3 (Fig. 1; Fig. 2). Beyond those, combined with high levels of starch and soluble protein contents in the leaves of soybean cv. Xiangdou No. 3 under the HTH stress (Fig. 3), our results indicated that the leaf of pre-harvest seed deterioration-resistant soybean cv. Xiangdou No. 3 had stronger photosynthesis and nutrient supply than that of pre-harvest seed deterioration-sensitive soybean cv. Ningzhen No. 1 under HTH stress.

In the pod (skin) cell, TEM analysis showed that all the organelles disappeared in soybean cv. Ningzhen No. 1 under the HTH stress, while the number of mitochondria was increased in cv. Xiangdou No. 3 (Fig. 2). Mitochondria is a principal source of ROS in plant cells, and is the early target of oxidative injuries, which can be accelerated to a greater degree than in other organelles during deterioration [38, 39]. The disappearance of organelles in the pod (skin) cell of soybean cv. Ningzhen No. 1 indicated that the cell ultrastructure was damaged under the HTH stress, whereas the cell ultrastructure in the pod (skin) cell of soybean cv. Xiangdou No. 3 was still maintained in a good condition. Moreover, a sharply increase in POD, CAT and SOD enzymes activities as well as high levels of starch, sucrose and soluble protein contents were found in the pod of soybean cv. Xiangdou No. 3 under the HTH stress (Fig. 3; Additional file 4: Fig. S4). These results indicated that the ROS scavenging and the nutrient transportation were enhanced in the pod of soybean cv. Xiangdou No. 3 under the HTH stress. In total, our results implied that the functions of pod in protection and nutrient supply for seeds in pre-harvest seed deterioration-resistant soybean cv. Xiangdou No.3 were still stronger than in pre-harvest seed deterioration-sensitive soybean cv. Ningzhen No. 1 under the HTH stress (Fig. 2; Fig. 3; Additional file 4: Fig. S4).

**Fig. 8:**
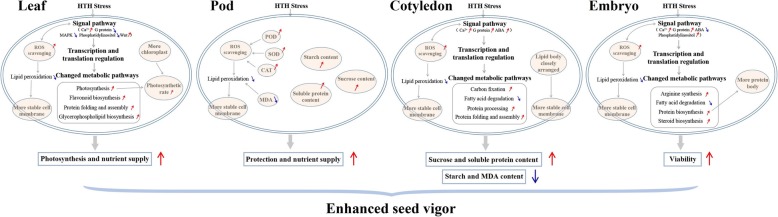
Graphic depiction for vigor enhancement mechanism in soybean under HTH stress. The enhanced metabolic pathways and cellular processes or the increased metabolites, in leaf, pod skin, cotyledon, and embryo are marked with “↑”, while the reduced marked with “↓”. ABA, abscisic acid; CAT, catalase; MAPK, mitogen-activated protein kinase; MDA, malondialdehyde; POD, peroxidase; ROS, reactive oxygen species; SOD, superoxide dismutase

Taken together, when the seed (cotyledon and embryo), leaf, and pod of soybean were exposed to HTH stress, their signaling pathways (ABA-mediated, MAPK, G protein- mediated, Ca^2+^-mediated, and phosphatidylinositol) were severely affected. Thereafter, some metabolic pathways and cellular processes (photosynthesis, glycolysis, protein biosynthesis, protein folding and assembly, oxidative stress defense) were enhanced to improve seed vigor, while some other metabolic pathways and cellular processes would be lowered to reduce seed vigor. Under the persistent HTH stress, the relatively enhanced function of cotyledon (the increase of sucrose and soluble protein contents, the decrease of starch and MDA contents and protein biosynthesis), embryo (viability), leaf (photosynthesis and nutrient supply), and pod skin (protection and nutrients supply) led to the improvement of seed vigor (Fig. 8). The HTH stress had less negative effects on the signal pathways, metabolic pathways, cell ultrastructure, and physiology and biochemistry in the cotyledon, embryo, leaf, and pod of pre-harvest seed deterioration-resistant soybean cv. Xiangdou No. 3 than on those of pre-harvest seed deterioration-sensitive soybean cv. Ningzhen No. 1, leading to a higher seed vigor in Xiangdou No. 3 (Fig. 4). Song et al. [23] suggested that the developing seed of cv. Xiangdou No. 3 possessed the greater ability of ROS scavenging, and cell rescue and defense than that of cv. Ningzhen No. 1 under HTH tress, which might be one of the major reasons why it was more deterioration-resistant than the latter. Thus, all these results will provide comprehensive characteristics of leaf, pod and seed under the HTH stress, which will be used as selection index in soybean breeding program for high seed vigor.

### Why the seeds produced from the HTH stressed plants could still germinate right?

After 168 h of HTH stress, the plants of the two soybean cultivars was still alive, but the leaves were aged, and the photosynthetic capacity was basically lost (Fig. 1). Why the seeds produced from the stressed plants could still germinate right (Fig. 4)? The reasons might be as follows: firstly, the stressed time for the plants was chosen at physiological maturity (R7 period) when the seeds started to possess the capability of germination. The HTH stress of 168 h in the present study was incapable of causing seeds to completely lose the ability of germination. Secondly, when the leaves were being aged, their main nutrients were quickly transported to the seeds, guaranteeing seed development and maturity (Fig. 3). Thirdly, the pod would provide the protection for seeds. In addition, the seed itself had the mechanism of protection and repair under stress environment. However, there are great differences among soybean cultivars in resistance to HTH stress.

### The comparison between the HTH stress and the controlled deterioration treatment (CDT)

CDT is widely used as a vigor assay for numerous seed species [10, 18, 20]. The harvested seeds are firstly subjected by CDT before the standard germination test. However, the HTH stress treatment in the present study is used to investigate its effects on the seed growth and development as well as germination at R7 period. Many studies have shown that CDT can affect the seed viability of *Arabidopsis*, *Brassica napus*, *Oryza sativa* and *Glycine max* [10, 18, 20, 40]. Some metabolic pathways were found to be changed in CDT and HTH treatments. For example, HTH stress enhanced the accumulation of GAPDH in cotyledon of pre-harvest seed deterioration-resistant soybean cv. Xiangdou No. 3 (Fig. 7; Additional file 10: Table S10), whereas a decrease in GAPDH level was found in aged *Arabidopsis* and wheat seeds through CDT [10, 41]. Zhang et al. [19] reported that rice seed aging (through CDT) was associated with increased abundance of ADH in the embryo, whereas the similar result was found in cotyledon between the two soybean cultivars under HTH stress in this study (Fig. 7). All the results of our research and Rajjou et al. [10] indicated that cysteine synthesis was an important feature of germination potential. Yin et al. [18] reported the involvement of ABA in the initiation of seed aging. Interestingly, in this study, compared to soybean cv. Ningzhen No. 1, the abscisic acid signaling was increased in cotyledon of cv. Xiangdou No. 3 under the HTH stress, but decreased in embryo.

## Conclusion

The HTH stress affected seed vigor through the negative effects on the signal pathways (ABA-mediated, MAPK, G protein-mediated, Ca^2+^-mediated, and phosphatidylinositol), metabolic pathways (photosynthesis, glycolysis, protein biosynthesis, protein folding and assembly, oxidative stress defense), cell ultrastructure, and physiology and biochemistry (antioxidases activities, sucrose, starch and soluble protein contents) in leaf, pod, cotyledon, and embryo of soybean. Soybean cultivars more tolerant to HTH stress produce higher vigor seeds.

## Methods

### Planting and sampling

Pre-harvest seed deterioration-resistant cv. Xiangdou No. 3 and -sensitive cv. Ningzhen No. 1, which were previously screened out by incubator weathering followed by standard germination test, were used in this study [21]. Soybean cvs. Xiangdou No. 3 and Ningzhen No. 1 were two extension cultivars in South China. Soybean cv. Xiangdou No. 3 was bred by Hunan Academy of Agricultural Sciences, while Soybean cv. Ningzhen No. 1 was bred by Jiangsu Academy of Agricultural Sciences in China. Seedlings of the two cultivars were grown in plastic pots. The plants were divided into two groups when they reached the physiological maturity period (R7). The stressed group was evenly transferred to three independent growth chambers [40 °C/30 °C, 100%/70% humidity, and 10 h/14 h cycle (day/night)] for 7 d. The control group with the same developmental progression were also placed in three separate chambers under 30 °C/20 °C, 70% humidity, and 10 h/14 h (day/night). The seeds (cotyledons and embryos), leaves and pods in the middle of the plants (10 plants) were sampled at 24, 96, and 168 h from each chamber. Therefore, each treatment or control has three biological replicates. The embryos stripped carefully from sample seeds by blade. Collected samples were frozen in liquid nitrogen immediately and stored at − 80 °C until use.

### Photosynthesis measurement

Leaf net photosynthetic rates were measured using a portable gas analysis system, LI-COR 6400 (Li-Cor Inc., Lincoln, NE, USA) according to [42]. The measurement conditions were set as follows: leaf temperature at 25 °C, 1000 μmol photons m^− 2^·s^− 1^. Each sample was measured for three times. All measurements were carried out between 9:00 am and 11:00 am to minimize the error.

### Transmission electron microscope analysis

TEM analysis was conducted according to [43]. Fresh leaves, pods, cotyledons and embryos from treatments and controls were cut into 2 mm × 3 mm, respectively, and fixed in a mixture of 2.5% glutaraldehyde in a 20 mM sodium cacodylate buffer (pH 7.0) for at least 12 h at 4 °C, and then postfixed with 1% KMnO_4_ for 2 h. These fixed samples were dehydrated in an ethanol series and embedded in Spurr resin. Ultrathin sections (70 nm thick) were stained with uranyl acetate and lead citrate. Observations were made on an H-7650 (Tokyo, Japan) TEM. Construction of high-resolution TEM pictures was carried out as described by [44].

### Enzyme activity assays

Soybean leaves, pods, cotyledons and embryos (0.5 g) from treatments and controls was ground, respectively, in a mortar with 1 ml of chilled 0.1 M phosphate buffer (pH 7.5) containing 1% (w/v) polyvinylpolypyrrolidone. Homogenates were centrifuged at 14,000×*g* at 4 °C for 30 min and the supernatants were used for enzyme activity assays. Five technical replicates were performed for each sample. Enzyme activities of CAT, POD and SOD were determined following the procedure described by [45]. All experiments were repeated for three times.

### Contents analysis of soluble protein, soluble sugar, MDA and starch

Soybean leaves, pods and cotyledons (0.5 g) from treatments and controls were used in this experiment. Concentration of the soluble protein was measured according to [46] with bovine serum albumin as standard. Content of soluble sugar was determined as previously described in [47]. The level of lipid peroxidation was determined as described in [48]. The total starch content was analyzed according to the method of [49]. All experiments were repeated for three times.

### Germination test

A series of germination tests were performed at 24, 96 and 168 h after HTH stress, respectively, according to [50]. Three replications (50 seeds for each treatment) were distributed in plastic boxes (20 × 11.5 × 8 cm) containing six sheets of moistened filter paper. Plastic boxes were put in a germination chamber at 20 °C, 60% humidity, 8 h light and 16 h darkness. Normal seedlings were recorded every day following incubation until 7 days, germination potential, germination rate and seedling height were determined, germination potential was assessed by the germination rate of the seeds on the fourth day.

### Protein extraction, digestion and labeling

Protein extraction was conducted as reported by [51]. The protein concentration was determined by the method of Bradford [46] using bovine serum albumin as standard. For each sample, an aliquot (100 μg) of protein was reduced with 10 mM dithiothreitol for 1 h at 37 °C and alkylated with 20 mM iodoacetamide for 1 h at room temperature in dark. Protein samples were digested using an enzyme to substrate ratio of 1:20 sequencing-grade trypsin (Promega, Madison, WI) at 37 °C overnight and then resultant peptide mixture was labeled using chemicals from iTRAQ reagent kit (Applied Biosystems, California, USA). All the iTRAQ labels were shown in Additional file 11: Table S11. The changes of protein level within cultivar at different time points were determined through comparison of point to point between the HTH treatment and the control in the same seven-plex. To compare the changes of protein level between the two cultivars under HTH stress, first, all the protein level data were normalized through comparison with the mixed sample from cv. Ningzhen No. 1 and cv. Xiangdou No. 3 at 24 h under the control condition in the same seven-plex; second, for each protein identified, its relative level ratios of cv. Xiangdou No. 3 to cv. Ningzhen No. 1 under the HTH stress were calculated as follows.

$$ \mathrm{Relative}\ \mathrm{protein}\ \mathrm{level}\ \mathrm{ratio}=\left({\mathrm{X}}_{\mathrm{T}}\div {\mathrm{N}}_{\mathrm{T}}\right)\div \left({\mathrm{X}}_{\mathrm{C}}\div {\mathrm{N}}_{\mathrm{C}}\right) $$

Where, X_T_ and N_T_ indicate the relative level of a protein in cv. Xiangdou No. 3 and cv. Ningzhen No. 1 under HTH stress after normalization, respectively, while X_C_ and N_C_ in cv. Xiangdou No. 3 and cv. Ningzhen No. 1 under control condition after normalization, respectively.

### iTRAQ analysis

For each organ sample (leaf, cotyledon, and embryo) that was equal weight mixed sample of the organ from 10 plants, three iTRAQ biological repeats were conducted. Therefore, there were 27 groups (3 cultivars or cultivar combinations × 3 organs × 3 biological replicates) data in the present study (Additional file 11: Table S11). Each iTRAQ reagent was dissolved in 50 μl of isopropanol, which was added to the respective peptide mixture. The digested peptides were labeled with iTRAQ reagents following the manufacturer’s instructions (AB Sciex). Firstly, the peptides were fractionated on a waters UPLC using a high pH C18 column (Waters Bec C18, 1.7 μm, 2.1 mm × 50 μm), then the fractionations were analyzed by nano-HPLC on the secondary reverse phase analytical column (Eksigent, C18, 3 μm, 150 mm × 75 μm). Peptides were eluted using a linear gradient, starting from 5 to 45% buffer B in 70 min (buffer A, 98% water with 0.1% formic acid, buffer B, 98% acetonitrile with 0.1% formic acid). The total flow rate was maintained at 300 nL/min. Electrospray voltage of 2.3 kV versus the inlet of the mass spectrometer was used. TripleTOF 5600 mass spectrometer was operated in data-dependent mode to switch automatically between MS and MS/MS acquisition. MS spectra were acquired across the mass range of 350–1250 m/z in high resolution mode using 250 ms accumulation time per spectrum. Tandem mass spectral scanned from 100 to 1250 m/z in high sensitivity mode with rolling collision energy. The 20 most intense precursors were selected for fragmentation per cycle with dynamic exclusion time of 9 s.

### Mass spectra data and protein quantification

Maxquant software v. 1.5.2.8 was used for large-scale tandem mass spectrometry data analysis [52]. The *Glycine max* UniProtKB/Swiss-Prot database were used, and downloaded on December, 2016 with 124,278 sequences. The wiff files generated by the TripleTOF 5600 instrument were searched directly using a 20 ppm precursor mass tolerance and a 50 mmu fragment mass tolerance. All the peptides FDR were dynamically set as 1%, which were calculated by a decoy database search (using a reverse sequence version of the reference database), and each confident protein included at least one unique peptide [53]. Redundancy between the annotated proteins and proteins identified from translation of the soybean genome was identified using BLAST [54], and all the identified proteins was list in Additional file 12: S1-S9. Screening criteria for DAPs were as follows: the proteins with a t-test *p*-value < 0.05 for two compared groups with three replicates, peptides ≥2 and a protein ratio > 1.5 or < 0.67-fold change [55].

### Bioinformatics analysis

The functional analysis of DAPs was conducted using Gene Ontology (GO) (http://www.ebi.ac.uk/QuickGO/) [56]. The DAPs were further assigned by the KEGG (Kyoto Encyclopedia of Genes and Genomes) database (http://www.kegg.jp/kegg/pathway.html) [57].

### Quantitative RT-PCR analysis

The expressions of eight genes encoding candidate HTH-responsive proteins were analyzed by qRT-PCR. These HTH-responsive proteins were differentially accumulated in the seeds between soybean cv. Xiangdou No. 3 and cv. Ningzhen No. 1. Total RNA was extracted from seeds, and then qRT-PCR was performed according to protocol of Liu et al. [58]. The primers were listed in Additional file 13: Table S13. The relative quantification (2^-ΔΔCt^) of gene expression was evaluated using comparative cycle threshold method, and each sample was replicated for three times.

### Statistical analysis

The *t*-test was used for pair-wise comparison of proteomic data and analysis of the significant changes in physiological data (SPSS 19.0, IBM, USA) with a confidence interval of 95% or 99%.

## Supplementary information

**Additional file 1 **: **Figure S1**. Enzyme activities of CAT, POD, and SOD in the cotyledons of soybean cvs. Xiangdou No. 3 and Ningzhen No. 1

**Additional file 2 **: **Figure S2**. Enzyme activities of CAT, POD, and SOD in the embryos of soybean cvs. Xiangdou No. 3 and Ningzhen No. 1

**Additional file 3 **: **Figure S3**. Enzyme activities of CAT, POD, and SOD in the leaves of soybean cvs. Xiangdou No. 3 and Ningzhen No. 1

**Additional file 4 **: **Figure S4**. Enzyme activities of CAT, POD, and SOD in the pod skin of soybean cvs. Xiangdou No. 3 and Ningzhen No. 1

**Additional file 5 **: **S1- S6**. Differentially abundant proteins in cotyledons, embryos, and leaves between the stressed and control in soybean cvs. Ningzhen No. 1 and Xiangdou No. 3, respectively. **S7- S9**. Differentially abundant proteins in cotyledons, embryos, and leaves between the soybean cv. Ningzhen No. 1 and cv. Xiangdou No. 3 under HTH stress

**Additional file 6 **: **S1- S9**. GO and KEGG analysis of the differentially abundant proteins in cotyledons, embryos, and leaves

**Additional file 7 **: **Figure S7**. Go classification of differentially abundant proteins identified in cotyledons between the control and the stressed in soybean cvs. Ningzhen No. 1 (A) and Xiangdou No. 3 (B), respectively, and between both the cultivars under the HTH stress (C)

**Additional file 8 **: **Figure S8**. Go classification of differentially abundant proteins identified in embryos between the control and the stressed in soybean cvs. Ningzhen No. 1 (A) and Xiangdou No. 3 (B), respectively, and between both the cultivars under the HTH stress (C)

**Additional file 9 **: **Figure S9**. Go classification of differentially abundant proteins identified in leaves between the control and the stressed in soybean cvs. Ningzhen No. 1 (A) and Xiangdou No. 3 (B), respectively, and between both the cultivars under the HTH stress (C)

**Additional file 10 **: **Table S10**. Key KEGG pathways for seed vigor formation in leaves, cotyledons, and embryos between the stressed and control in soybean cvs. Ningzhen No. 1 and Xiangdou No. 3, respectively, and between both the cultivars

**Additional file 11 **: **Table S11**. 7-plex iTRAQ experimental design

**Additional file 12 **: **S1-S9**. The list of all the identified proteins

**Additional file 13 **: **Table S13**. Sequences of primers used in this study

## Data Availability

The data sets supporting the conclusions of this article are included within the article and its additional files. The mass spectrometry proteomic data have been submitted to a public iProX database (https://www.iprox.org/page/PSV023.html;?url=1578575601558rHFc), the password is I9ux.

## Abbreviations

ABA: Abscisic acid
ADH: Alcohol dehydrogenase
CAT: Catalase
CCO: Cytochrome c oxidase
CDT: Controlled deterioration treatment
CP: Chaperone protein
CV: Cultivar
DAP: Differentially abundant protein
DHD: Dehydrin
GAPDH: Glyceraldehyde-3-phosphate dehydrogenase
GO: Gene Ontology
HSP: Heat shock protein
HTH: High temperature and humidity
iTRAQ: Isobaric tags for relative and absolute quantification
KEGG: Kyoto Encyclopedia of Genes and Genomes
LPX: Lipoxygenase
MAPK: Mitogen-activated protein kinase
MDA: Malondialdehyde
MAS: Mitochondrial ATP synthase subunit O
MT: 5-methyltetrahydropteroyltriglutamate--homocysteine methyltransferase
MS: Methionine synthase
POD: Peroxidase
ROS: Reactive oxygen species
RP: Ribosomal protein
RuBisCO: Ribulose bisphosphate carboxylase small chain
SHSP: Small heat shock protein
SOD: Superoxide dismutase
STI: Heat shock protein STI
TCA: Tricarboxylic acid
TEM: Transmission electron microscope
