# Supplementary material for: Quantitative proteomic, physiological and biochemical analysis of cotyledon, embryo, leaf and pod reveals the effects of high temperature and humidity stress on seed vigor formation in soybean
Source: BMC Plant Biol. 2020 Mar 26;20:127. doi: 10.1186/s12870-020-02335-1 (PMC7098090; doi:10.1186/s12870-020-02335-1)
Supplement: Supplementary file 4 — Additional file 4 : Figure S4. Enzyme activities of CAT, POD, and SOD in the pod skin of soybean cvs. Xiangdou No. 3 and Ningzhen No. 1 [file 12870_2020_2335_MOESM4_ESM.docx]

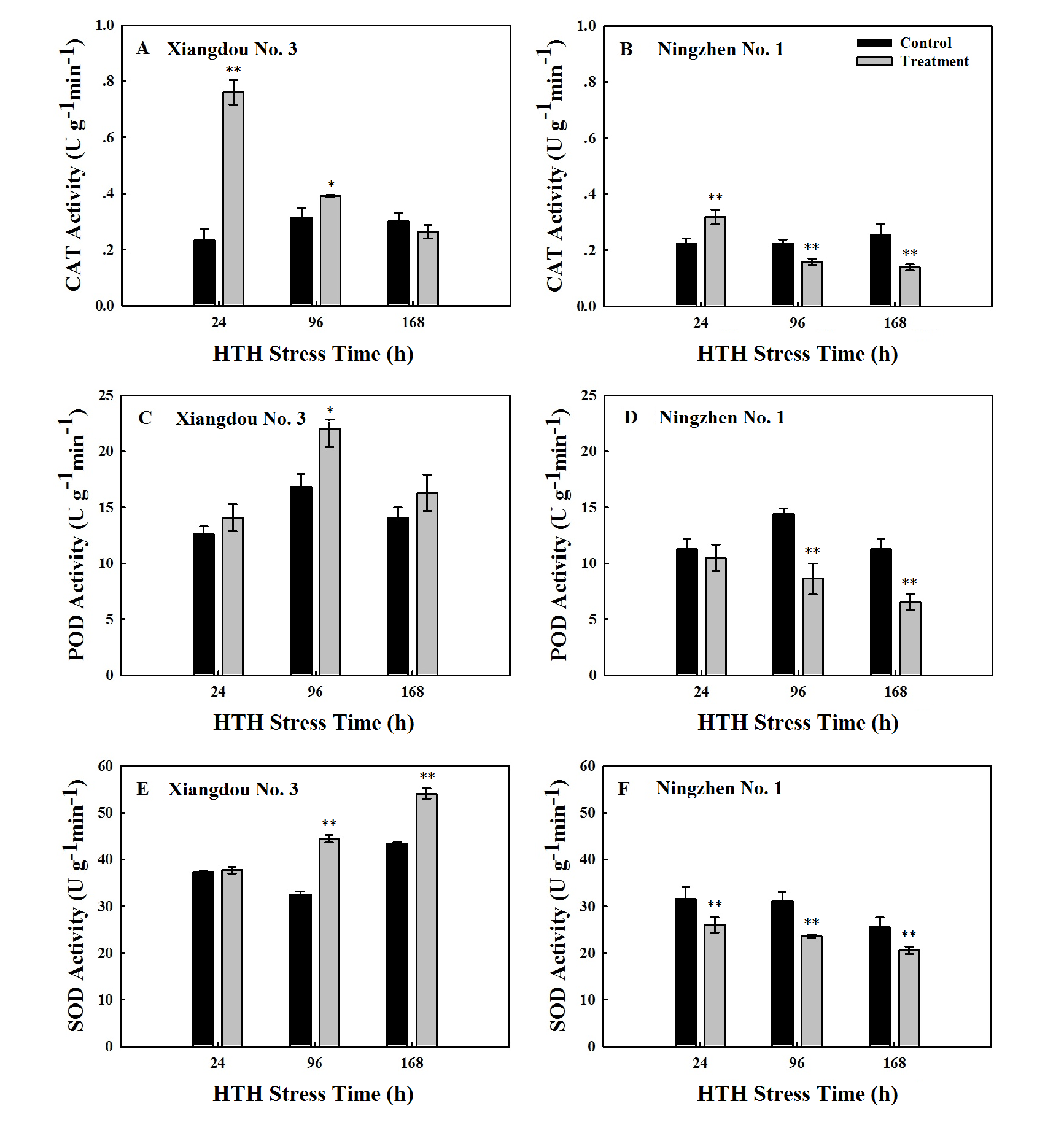


**Additional files 4: Fig S4. Enzyme activities of CAT, POD, and SOD in the pod skin of soybean cvs. Xiangdou No. 3 and Ningzhen No. 1.**

**A**, **C**, **E**, The controls and treatments of cv. Xiangdou No. 3; **B**, **D**, **F**, The controls and treatments of cv. Ningzhen No. 1**.** Values shown are means ± SD from three biological replicates (**, *p* < 0.01; *, *p* < 0.05).
