# Supplementary material for: Quantitative proteomic, physiological and biochemical analysis of cotyledon, embryo, leaf and pod reveals the effects of high temperature and humidity stress on seed vigor formation in soybean
Source: BMC Plant Biol. 2020 Mar 26;20:127. doi: 10.1186/s12870-020-02335-1 (PMC7098090; doi:10.1186/s12870-020-02335-1)
Supplement: Supplementary file 8 — Additional file 8 : Figure S8. Go classification of differentially abundant proteins identified in embryos between the control and the stressed in soybean cvs. Ningzhen No. 1 (A) and Xiangdou No. 3 (B), respectively, and between both the cultivars under the HTH stress (C) [file 12870_2020_2335_MOESM8_ESM.docx]

**
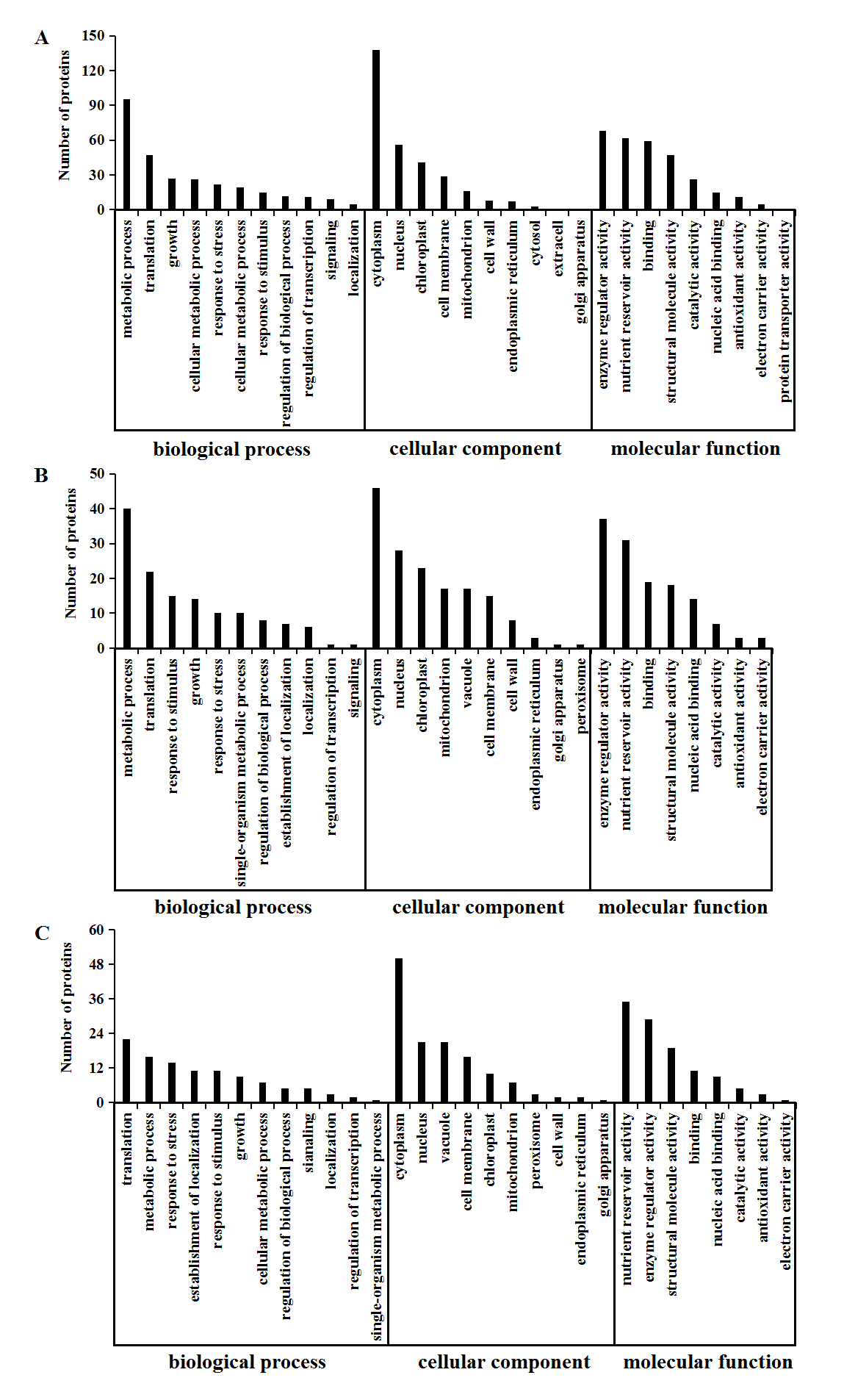
**

**Additional files 8: Fig S8. Go classification of differentially abundant proteins identified in embryos between the control and the stressed in soybean cvs. Ningzhen No. 1 (A) and Xiangdou No. 3 (B), respectively, and between both the cultivars under the HTH stress (C).**
